# A Minimally-invasive Blood-derived Biomarker of Oligodendrocyte Cell-loss in Multiple Sclerosis

**DOI:** 10.1016/j.ebiom.2016.06.031

**Published:** 2016-06-27

**Authors:** John A. Olsen, Lauren A. Kenna, Regine C. Tipon, Michael G. Spelios, Mark M. Stecker, Eitan M. Akirav

**Affiliations:** a Research Institute, Islet Biology, Winthrop-University Hospital, Mineola, NY, USA; b Department of Neuroscience, Winthrop-University Hospital Mineola, NY, USA; c Stony Brook University School of Medicine, Stony Brook, NY, USA

**Keywords:** Relapsing-remitting multiple sclerosis, Oligodendrocyte, Cuprizone, Human patients, Circulating free DNA, Biomarker discovery

## Abstract

Multiple sclerosis (MS) is a neurodegenerative disease of the central nervous system (CNS). Minimally invasive biomarkers of MS are required for disease diagnosis and treatment. Differentially methylated circulating-free DNA (cfDNA) is a useful biomarker for disease diagnosis and prognosis, and may offer to be a viable approach for understanding MS. Here, methylation-specific primers and quantitative real-time PCR were used to study methylation patterns of the myelin oligodendrocyte glycoprotein (MOG) gene, which is expressed primarily in myelin-producing oligodendrocytes (ODCs). MOG-DNA was demethylated in O4^+^ ODCs in mice and in DNA from human oligodendrocyte precursor cells (OPCs) when compared with other cell types. In the cuprizone-fed mouse model of demyelination, ODC derived demethylated MOG cfDNA was increased in serum and was associated with tissue-wide demyelination, demonstrating the utility of demethylated MOG cfDNA as a biomarker of ODC death. Collected sera from patients with active (symptomatic) relapsing-remitting MS (RRMS) demonstrated a higher signature of demethylated MOG cfDNA when compared with patients with inactive disease and healthy controls. Taken together, these results offer a minimally invasive approach to measuring ODC death in the blood of MS patients that may be used to monitor disease progression.

## Highlights

• Currently, there are no molecular biomarkers of multiple sclerosis (MS).
• A minimally invasive assay for measuring oligodendrocyte (ODC) cell loss in relapsing-remitting MS is described.
• DNA methylation of the myelin oligodendrocyte glycoprotein gene is used as a measure of ODC loss in the blood.

Multiple sclerosis (MS) is a neurodegenerative disease of the central nervous system. Currently, there are no molecular biomarkers of MS, thereby limiting disease diagnosis, prognosis, and assessment of new clinical interventions. Myelin oligodendrocyte glycoprotein (MOG) is expressed solely by oligodendrocytes (ODCs) as an integral part of the myelin sheath. This report describes a minimally invasive biomarker assay for measuring ODC-derived DNA in the blood of MS patients. It describes the presence of unique DNA methylation patterns in the MOG gene in ODCs, which is used to design methylation-specific primers. Analysis of sera from patients with active relapsing-remitting MS shows an increase in levels of ODC-derived circulating free DNA when compared with inactive disease and healthy controls.

## 1 Introduction

Multiple sclerosis (MS) is a neurodegenerative disease characterized by demyelination of axons in the central nervous system (CNS). The cells that produce myelin in the CNS, oligodendrocytes (ODCs) (Bunge et al., 1962, Bunge, 1968), die from an autoimmune response that results in demyelinated lesions in the brain and spinal cord (Noseworthy et al., 2000, Olsen and Akirav, 2015). Axonal demyelination reduces the signal strength of nerve impulses (Waxman, 1977, Felts et al., 1997) and may leave the axon exposed to degeneration (Ferguson et al., 1997). Demyelinated lesions can be visualized by magnetic resonance imaging (MRI); however, this procedure is costly and cannot detect diffused or mild myelin degeneration or measure the active loss of ODCs. Although new innovations in MS therapy are currently being explored (Olsen and Akirav, 2015), there are currently no clinically approved molecular biomarkers of ODC death in MS. This unmet need impairs MS diagnosis, prognosis, and assessment of clinical intervention.

DNA methylation is a mechanism involved in the control of tissue-specific gene expression (Jones, 2012). DNA hypermethylation of CpG dinucleotides acts as an inhibitor of transcription while hypomethylation is associated with increased gene expression. Differences in DNA methylation patterns between different cell types can be used to determine the origin of the DNA. Accordingly, methylated circulating-free DNA (cfDNA) is used as a biomarker of tumor progression (Nawroz et al., 1996, Chen et al., 1996, Crowley et al., 2013). Our group has successfully adapted this approach for the detection of β-cell loss in patients with autoimmune type 1 diabetes (Akirav et al., 2011), where differentially methylated β-cell derived insulin cfDNA was used to detect β-cell loss. A recent report by Lehmann-Werman et al. showed the utility of using cfDNA to detect cell loss in MS, by measuring demethylated cfDNA of MBP and WM1 genes, showing an increase in the levels of demethylated DNA in patients with MS (Lehmann-Werman et al., 2016).

In contrast to MBP, which is found in both ODCs and Schwann cells, and WM1, whose tissue expression patterns are largely unknown, myelin oligodendrocyte glycoprotein (MOG) is a CNS-specific protein expressed solely by ODCs as an integral part of the myelin sheath (Linnington et al., 1984, Lebar et al., 1986, Gardinier et al., 1992, Johns and Bernard, 1999). The specificity of MOG in ODCs suggests that the MOG gene may present with unique methylation patterns in ODCs, which can be used to detect ODC-derived DNA in the blood and assess disease activity in patients with MS. Here we identify demethylated CpG dinucleotides in ODCs from mouse and human origin that are absent in other cell types in the brain and periphery. Methylation-specific primers for demethylated MOG-DNA were able to detect ODC-specific DNA in purified primary mouse ODCs and primary human oligodendrocyte precursor cells (OPCs). When used to detect ODC MOG-DNA in the sera, these primers showed an increase in ODC-derived cfDNA in mice treated with cuprizone. Testing of human primers successfully detected ODC MOG cfDNA in sera of patients with relapsing-remitting MS (RRMS) and showed a correlation with disease activity.

## 2 Methods

### 2.1 Mice

C57BL/6 female mice purchased from Jackson Laboratory (Bar Harbor, ME) were started on a diet of rodent chow with 0.2% cuprizone (Research Diets Inc., New Brunswick, NJ) at 8 weeks of age (Day 0). Mice were divided into 2 groups of 6 (total *n* = 12) with blood sampling by cheek pouch bleed on Days 0, 7, 21, 35, and 49 for group 1 and Days 0, 14, 28, and 42 for group 2. On Day 56 blood was obtained from all mice via heart puncture. Brain tissues were collected for histological analysis of myelination. All animal care was approved by the Winthrop-University Hospital Institutional Animal Care and Use Committee.

### 2.2 Cell and Cell Lines

SW10 immortalized neuronal murine Schwann cells were purchased from American Type Culture Collection (Manassas, VA) and cultured using the provided protocols. Cells were pelleted for DNA extraction. Human OPC genomic DNA extracts (ScienCell Research Laboratories, Carlsbad, CA) were used as a positive control sample for demethylated MOG-DNA. This genomic DNA sample was obtained from ScienCell Research Laboratories from early passage human OPCs using the AllPrep DNA/RNA Mini Kit (Qiagen N.V., Valencia, CA).

### 2.3 Mouse Tissues

Mouse liver, kidney, and brain tissue were obtained from a C57BL/6 mouse (Jackson Laboratory, Bar Harbor, ME) and homogenized for DNA extraction.

### 2.4 Enrichment of O4^+^ Cells

O4^+^ cells were purified from the brains of five C57BL/6 mice (Jackson Laboratory, Bar Harbor, ME) and treated with the Neural Dissociation Kit (P) and Anti-O4 MicroBeads, followed by magnetic purification on the autoMACS Pro Separator (Miltenyi Biotec Inc., Auburn, CA).

### 2.5 FACS Staining

Evaluation of O4^+^ fraction purity was performed by fluorescence-activated cell sorting (FACS) analysis. Magnetically purified ODCs were washed twice and suspended in FACS buffer containing anti-O4 labeled antibody or isotype control and analyzed using an Accuri FACS analyzer (BD Biosciences, San Diego, CA). FACS data was analyzed using FlowJo software (FlowJo, Ashland, OR).

### 2.6 Plasmid Injections

Four C57BL/6 mice (Jackson Laboratory, Bar Harbor, ME) were each administered an intravenous injection of TOPO plasmid containing the demethylated mouse MOG sequence. Five minutes following injection, blood was obtained by heart puncture.

### 2.7 Histology

Brain tissues from cuprizone or sham treated mice were collected immediately following euthanasia, embedded in O.C.T. compound (Fisher, Waltham, MA) and snap frozen in liquid nitrogen. Tissues were sectioned at 7 μm thickness and mounted on poly-d-lysine coated slides, followed by formalin fixation. Myelin staining was done using a NovaUltra Luxol Fast Blue Staining Kit according to the manufacturer's instructions (IHCworld, Woodstock, MD). Stained slides were imaged using a bright field on a Nikon Eclipse Ti confocal microscope (Nikon, Melville, NY).

### 2.8 Human Subject Samples

Serum samples were obtained from BioServe Biotechnologies, Ltd. (Beltsville, MD). Samples were designated as “Active” if having a relapse at the time of sampling, “Inactive” if in remission at the time of sampling, or “Healthy Control” based on donor information. Disease activity was determined by specialized neurologists at the time of sampling and included a combination of disease activity by patient admission, EDSS scoring and MRI imagining where applicable. In some cases, MS activity was determined primarily by physician assessment and patient reports. Demographic information, disease activity, disease duration, duration from the last episode, and time between episodes are summarized in Table 3.

### 2.9 DNA Purification and Bisulfite Conversion

DNA extraction from homogenized tissue, sera, and cell pellets was performed using the DNEasy Blood and Tissue Kit (Qiagen N.V., Valencia, CA). DNA concentration was measured with the Quant-iT PicoGreen dsDNA Assay Kit (Life Technologies, Carlsbad, CA). DNA extracts were bisulfite treated with the EZ DNA Methylation-Direct Kit (Zymo Research, Irvine, CA).

### 2.10 Bisulfite Sequence Prediction and Primer Design

Prediction of methylated CpG in specific regions of the human and mouse MOG gene was done using the MethPrimer program (Li and Dahiya, 2002). Sequence output was used to design methylation insensitive and sensitive primers.

### 2.11 First-step PCR and Gel Extraction

A first-step PCR using non-methylation-specific primers and EpiTaq HS Kit (Clonetech Laboratories Inc., Mountain View, CA) was run to increase template availability. First-step products were run on a 2% agarose gel and isolated using the QIAquick Gel Extraction Kit (Qiagen N.V., Valencia, CA). No template controls in the first-step reaction were free of products. Mouse and human primer sequences are described in Table 1, Table 2, respectively.

### 2.12 Cloning of MOG-DNA

Bisulfite treated first-step PCR products purified from agarose gel were used as templates for a TOPO TA cloning reaction using the pCR 2.1TOPO vector (Invitrogen, Carlsbad, CA). ODC + and liver first-step MOG products were used as the mouse demethylated and methylated inserts, respectively. Human demethylated and methylated DNA (Zymo Research, Irvine, CA) first-step MOG products were used for the human demethylated and methylated inserts, respectively. NEB 5-alpha competent *Escherichia* *coli* (New England Biolabs, Ipswich, MA) were used for transformation with the TOPO MOG products and incubated at 37 °C overnight on ImMedia Kan Agar plates (Invitrogen, Carlsbad, CA). Individual colonies were added to a culture of LB broth/Kanamycin (Gibco, Carlsbad, CA) and shaken at 37 °C overnight. The QIAprep Spin Miniprep Kit (Qiagen N.V., Valencia, CA) was used to purify the TOPO plasmid from the cultures.

### 2.13 McrBC Restriction Enzyme Reaction

Purified DNA from human liver, brain, and spinal cord fraction was treated with the McrBC methylation-specific restriction enzyme (New England Biolabs Inc., Ipswich, MA). After treatment, 70 ng of treated and untreated liver, spinal cord or brain DNA, as well as a TOPO plasmid containing the appropriate native MOG insert, were run on PCR using native MOG primers. Samples were run on PCR and were removed at cycle 32 or 35. PCR products were run on a 2% agarose gel and imaged using a 4000R Image Station (Eastman Kodak Co., Rochester, NY).

### 2.14 Sequencing of MOG-DNA

MOG first-step PCR gel extracts and MOG Plasmid DNA were sequenced at the Keck Biotechnology Research Laboratory (New Haven, CT).

### 2.15 Analysis of MOG Methylation in Primary Murine O4^+^ and O4^−^ Fractions

First-step PCR product of bisulfite treated DNA from mouse O4^+^ and O4^−^ fractions, SW10 Schwann cells, and liver were cloned using pCR 2.1TOPO vector (Invitrogen, Carlsbad, CA). NEB5-alpha competent *E. coli* (New England Biolabs, Ipswich, MA) were used for the transformation reaction and individual colonies were treated with the Zymo Zyppy-96 Plasmid Miniprep (Zymo Research, Irvine, CA) prior to sequencing. Purified clones were sequenced by the Keck Biotechnology Research Laboratory (New Haven, CT).

### 2.16 Methylation-sensitive Real Time PCR

Gel-extracted first-step PCR products served as a template for quantitative real-time PCR (qPCR) using methylation-sensitive primers (Sigma-Aldrich Corp., St. Louis, MO) designed for bisulfite treated MOG-DNA. All reactions were run using LightCycler 480 SYBR Green I Master (Roche Diagnostics GmbH, Germany) PCR mix on a CFX96 Real-Time System (Bio-Rad Laboratories Inc., Hercules, CA). Relative quantification of demethylated DNA was calculated using the demethylation index (DMI) = 2^(methylated cycle number) − (demethylated cycle number)^.

### 2.17 Statistical Analysis

Results are reported as mean ± SEM. Statistical significance (*p* < 0.05) of differences between means was calculated by one-way ANOVA and Tukey's post hoc test using Prism 5 (GraphPad software). In some cases, *t*-test was used to compare two individual treatment groups. ROC analysis of Active vs. Inactive RRMS was performed with Prism 5.

## 3 Results

### 3.1 MOG Gene DNA Shows Unique Methylation in the CNS

**Fig. 1:**
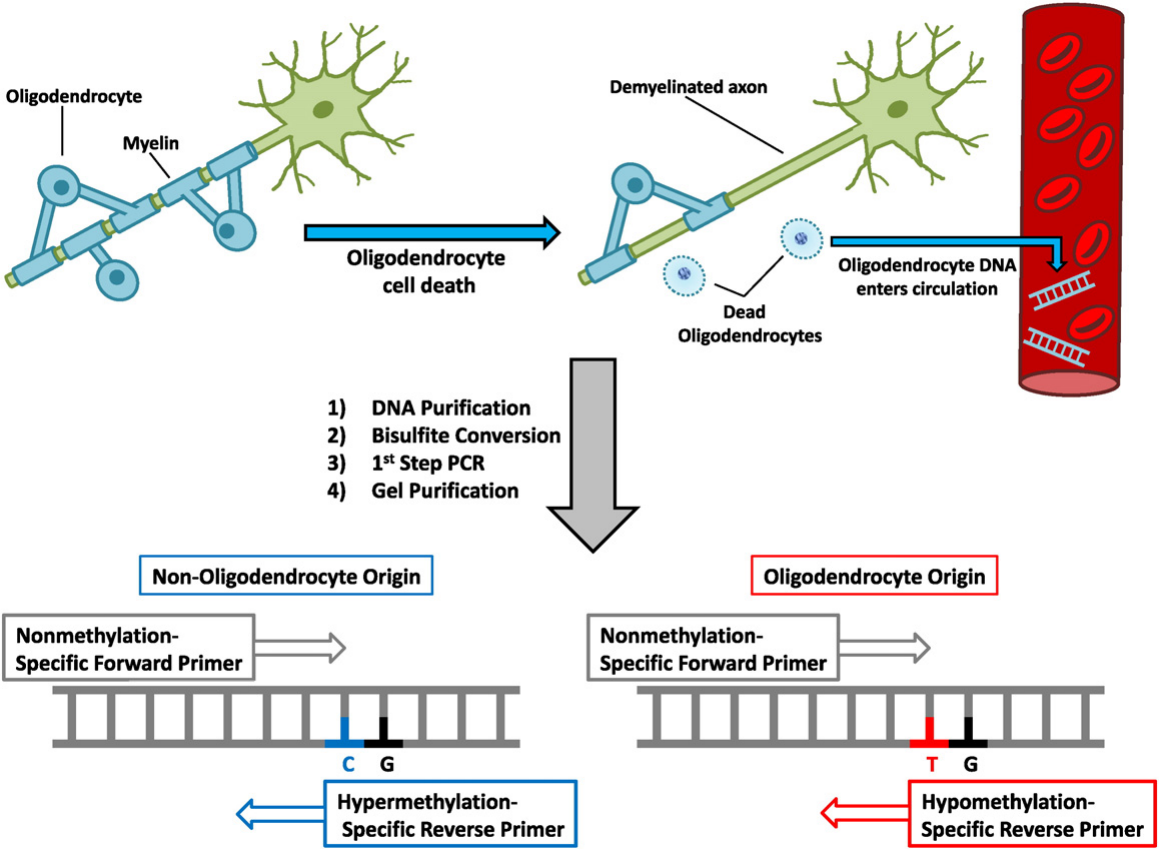
Illustrative representation of biomarker assay used to detect oligodendrocyte death in multiple sclerosis. Myelin-producing oligodendrocytes die and release genomic DNA into circulation. Blood is collected from the patient, DNA is purified, and bisulfite converted. Post-bisulfite conversion, samples are run on first-step PCR using methylation-unspecific primers and loaded onto an agarose gel. First-step PCR product is extracted and used as a template for qPCR utilizing methylation-specific primers.

**Fig. 2:**
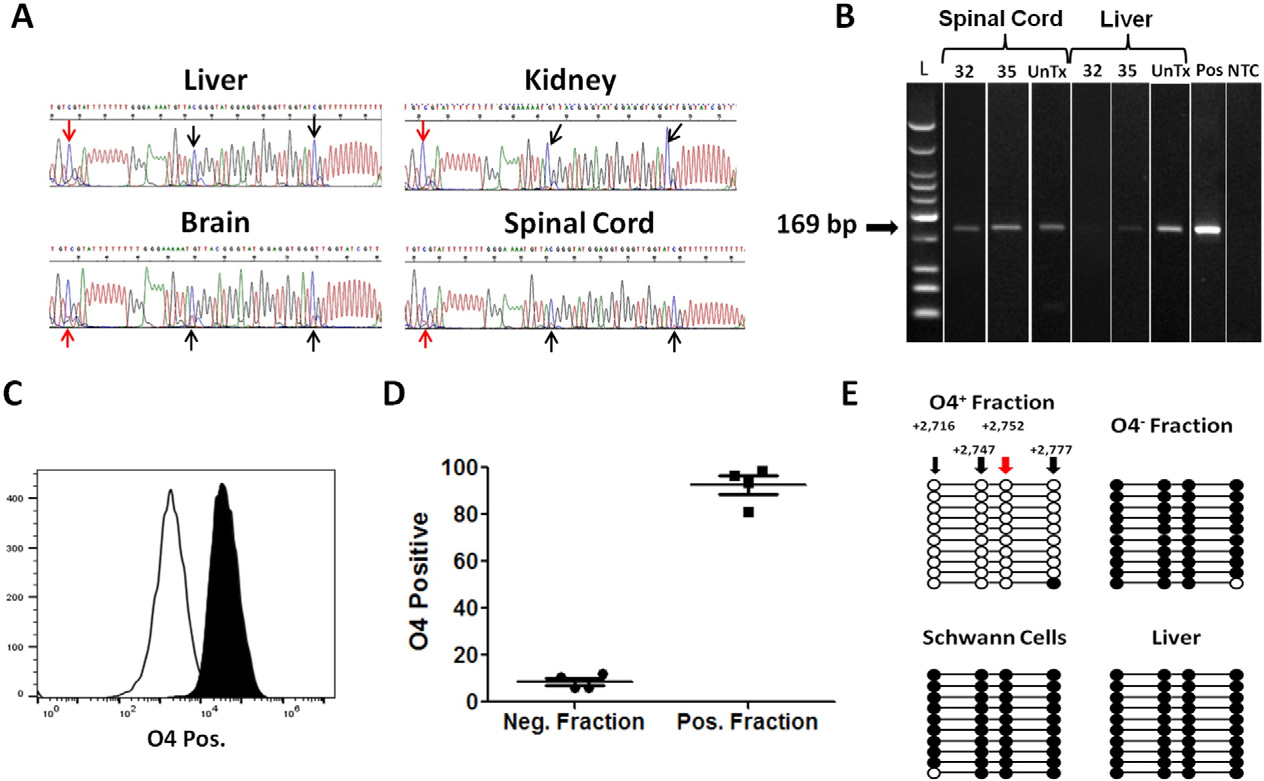
Murine brain and spinal cord show differential methylation in the MOG gene, due to their O4^+^ cell population. A. Sanger sequencing results of bisulfite treated DNA from murine tissues. Arrows point toward CpG sites where cytosines (C) are preserved in methylated samples (Liver, Kidney), or converted to thymines (T) in samples containing demethylated CpGs, leading to a mixed population of C′s and T's (Brain, Spinal Cord). B. Methylation-sensitive DNA digestion was performed on the magnetically spinal cord of liver derived-DNA using the McrBC enzyme. Digested DNA was subjected to semi-quantitative PCR (cycles 32 and 35) and run on agarose gel. UnTx—untreated DNA receiving all reaction components except McrBC enzyme. Pos—positive control using cloned native MOG DNA. NTC—no template control. C and D. O4^+^ and O4^−^ cells were separated from four separate murine brains by magnetic beads. FACS analysis showed > 92.6 ± 3.9% enrichment of O4^+^ cells among four independent preparations when compared to O4^−^ fractions. E. DNA from murine O4^+^ cells is differentially methylated in the MOG gene compared to DNA from O4^−^ cells, the SW10 Schwann cell line, and liver. Sequence analysis was performed on first-step PCR product of each sample, 10 clones from each are shown (○ represent demethylated cytosines; ●, methylated cytosines). Locations in relation to the MOG transcription start site are listed, methylation-specific murine primers incorporate the CpG site at bp + 2752.

The concept of using DNA methylation as a biomarker of ODC loss is described in Fig. 1. Previous reports show the expression of MOG in the brain, however, the role of epigenetics in controlling MOG expression remains largely unknown. To examine whether the MOG gene is demethylated in the CNS, DNA from murine liver, kidney, brain and spinal cord was isolated and subjected to bisulfite sequencing. Bisulfite treatment of DNA leads to the conversion of cytosine (C) to thymine (T) in demethylated (DeMeth) CpGs (C → T), while methylated (Meth) mCpGs remain intact (C → C). Sequence analysis of DNA from murine brain and spinal cord tissues revealed an increase in C → T conversion when compared with DNA from liver and kidney, demonstrating the presence of DeMeth CpGs in CNS tissue (Fig. 2A). Analysis of MOG DNA methylation in native DNA by the methylation sensitive restriction enzyme, MCRBC, confirmed the presence of demethylated MOG DNA in spinal cord DNA when compared with liver DNA (Fig. 2B). Similar results were obtained using brain DNA (data not shown). ODCs are considered the only source of MOG protein in the brain. Therefore, in order to confirm that MOG-DNA was indeed demethylated in ODCs and not in other cell types, brains from normal mice were digested and cells labeled with anti-O4 magnetic beads, followed by magnetic separation. FACS analysis of enriched O4^+^ and O4^−^ primary cell fractions were 92.6 ± 3.9% pure in four independent O4^+^ preparations when compared with the O4^−^ fraction (Fig. 2C and D). Purified O4^+^ and O4^−^ cells were lysed and genomic DNA purified and bisulfite converted. Analysis of MOG-DNA methylation state in both fractions revealed unique methylation patterns in O4^+^ cells but not in O4^−^ cells (Fig. 2E). Schwann cells mediate the myelination of peripheral nerves and are MOG negative. Analysis of the DNA methylation patterns in DNA from murine Schwann cell line and liver (negative control) showed a complete methylation of four CpG dinucleotides in MOG gene, demonstrating the fact that MOG demethylation is unique to ODCs (Fig. 2E and Supplemental Fig. 1). Taken together these data show demethylation patterns in the MOG-DNA from primary ODCs when compared with other CNS cell types, Schwann cells, and peripheral tissues, suggesting that these differences in methylation can be used as a biomarker of ODC loss.

### 3.2 Methylation Specific Primers Detect DeMeth MOG-DNA in Murine CNS Tissues and Purified ODCs

**Fig. 3:**
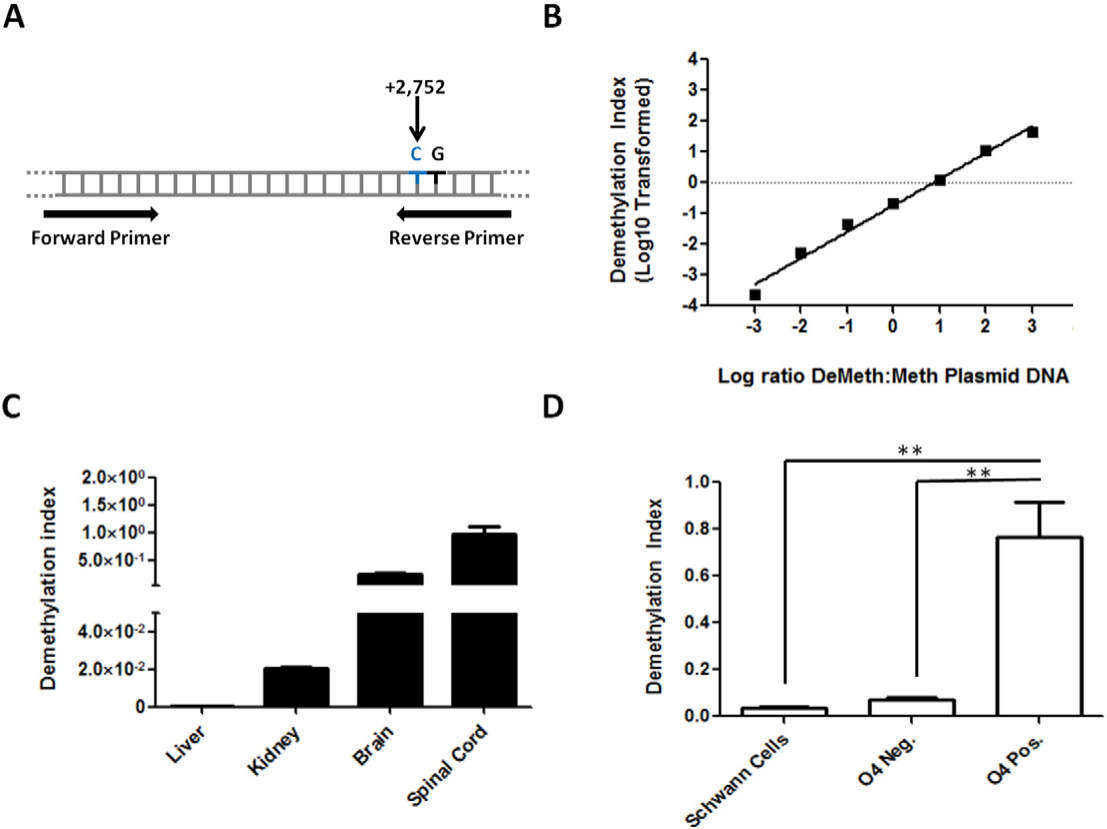
Methylation-specific primers display high specificity and sensitivity and can detect demethylated MOG-DNA in murine brain, spinal cord, and O4^+^ cells. A. A depiction of MOG gene region utilized for mouse methylation-specific qPCR analysis; cytosine at bp + 2752 from MOG transcription start site incorporated into reverse primer sequence. B. Methylation-specific primers tested using plasmids containing methylated and demethylated murine MOG-DNA inserts over a wide range of serial dilutions (R^2^ = 0.987, *p* < 0.0001). C. Methylation-specific primers were used in qPCR with bisulfite treated DNA from murine liver, kidney, brain, and spinal cord. Three independent analyses used to compute DMI averages; Liver vs. Brain/Spinal Cord *p* < 0.001, Kidney vs. Brain/Spinal Cord *p* < 0.001. D. Methylation-specific primers were used in qPCR with murine O4^+^ cells, O4^−^ cells, and SW10 Schwann cells. Three independent analyses used to compute DMI averages; ANOVA *p* = 0.0008, O4^+^ vs. O4^−^*p* < 0.01, O4^+^ vs. SW10 Schwann cells *p* < 0.01.

The identification of demethylated CpG dinucleotides in primary ODCs and CNS tissues prompted us to design methylation-specific primers capable of discerning between Meth and DeMeth CpGs in position + 2752 of the murine MOG gene (Table 1 and Fig. 3A). Plasmids containing the Meth and DeMeth DNA sequence of the MOG gene were used as controls for primer sensitivity and specificity. Serial dilution of plasmids over a 6 log dilution range showed the ability of DeMeth specific primers to detect DeMeth DNA even when diluted at 1:1000 in Meth-DNA (Fig. 3B, R^2^ = 0.987, *p* < 0.0001). When tested on bisulfite-treated DNA from murine tissues, methylation-specific primers showed a strong signal in both brain and spinal cord tissues when compared with liver and kidney DNA (Fig. 3C. ANOVA *p* < 0.0001. Liver vs. Brain or SC *p* < 0.001. Kidney vs. Brain or SC *p* < 0.001). Comparison of purified O4^+^ ODCs with both O4^−^ and SCW10 showed similar results, with O4^+^ cells producing a statistically higher signal when compared with either cell (Fig. 3D. ANOVA *p* = 0.0008, O4^+^ vs. O4^−^ *p* < 0.01. O4^+^ vs. Schwann cells *p* < 0.01). Taken together, these results show the ability of methylation-specific primers to identify DeMeth MOG-DNA in ODCs, suggesting that these primers may be used to detect ODC-derived cfDNA in the serum.

### 3.3 Circulating Free DeMeth MOG-DNA Is Detected in Sera of Mice with Widespread Demyelination

**Fig. 4:**
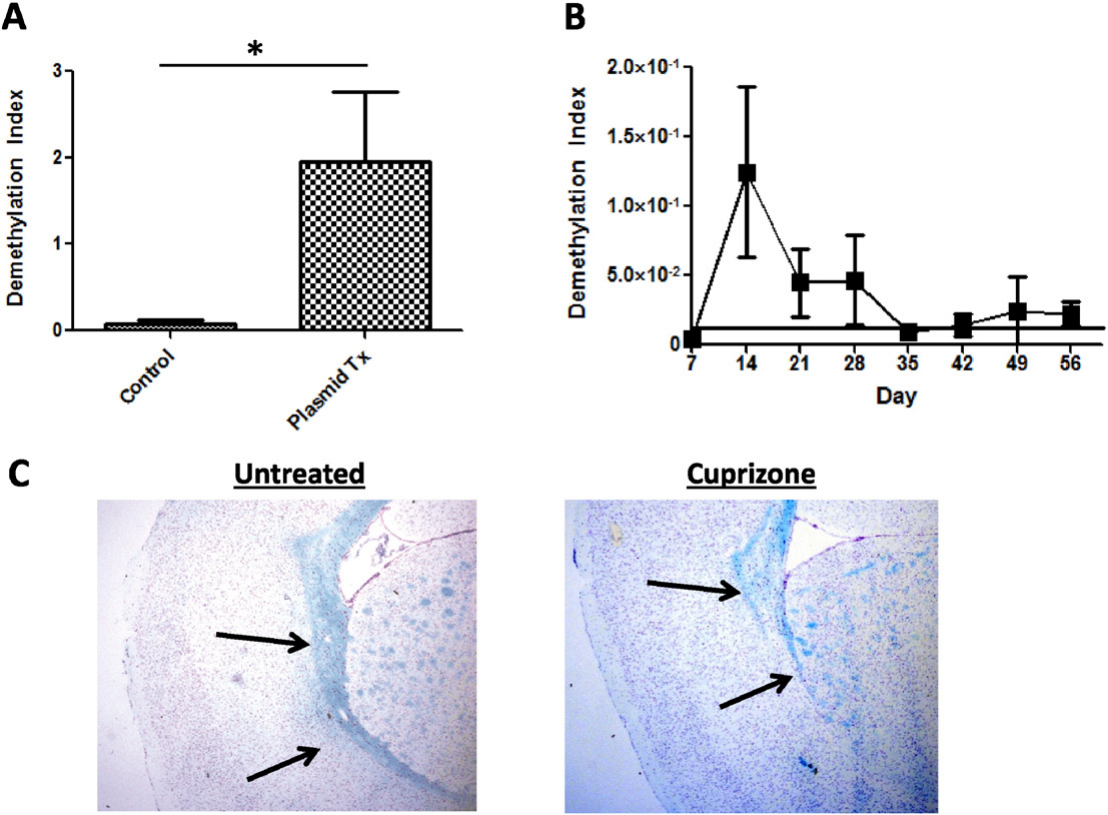
Methylation-specific primers can detect DeMeth MOG-DNA in the serum of plasmid injected mice and in cuprizone-treated mice which can be correlated with demyelination in neural tissue. A. Bisulfite-treated DNA from the serum of mice injected with DeMeth MOG plasmid and non-injected mice was run on qPCR with methylation-specific primers; Tx vs. Ctrl *p* < 0.012. B. Bisulfite-treated DNA from the serum of cuprizone-fed mice (*n* = 12) was run on qPCR with methylation-specific primers; MOG DMIs peak at Day 14 and remain elevated over baseline until Day 35. C. Brain sections from cuprizone-fed and control mice stained for myelin using Luxol fast-blue; arrows indicate the region of native myelination.

To examine whether DeMeth-specific primers can be used to detect DeMeth MOG in the blood, healthy C57BL/6 mice were injected with a plasmid containing an insert of DeMeth DNA of the MOG gene. Blood was collected 5 min post injection by heart puncture and circulating free serum DNA was purified. The level of DeMeth DNA in serum DNA was expressed as DMI. Extracted DNA from plasmid treated mice showed a 24-fold increase in DeMeth MOG-DNA when compared with untreated controls (Fig. 4A, Tx vs. Ctrl *p* < 0.012), demonstrating the ability of DeMeth-specific primers to detect DeMeth MOG-DNA in the serum. Next, we used the cuprizone mouse model of MS. In this model, ODC injury is induced by treatment with the copper chelating agent, cuprizone. Cheek pouch bleeding was performed on a weekly basis following cuprizone administration to measure the levels of ODC-derived DeMeth MOG cfDNA in the blood. Baseline DMI (Fig. 4B, solid line) was established using normal untreated mice (*n* = 9). Average MOG DMIs (*n* = 12, 6 mice per time point) reached their highest peak over baseline at Day 14, with increased DMI levels continuing through Days 21 and 28 before returning to baseline levels on Day 35 (Fig. 4B). Luxol fast-blue staining of brain tissue sections showed a gross demyelination in cuprizone-treated mice but not untreated mice, confirming the dramatic effect on myelin in the brain (Fig. 4C). These data demonstrate the ability of methylation-specific primers to detect DeMeth MOG-DNA in the blood of mice treated with the ODC-specific toxin, supporting the use of DeMeth MOG-DNA as a biomarker of ODC cell loss in MS.

### 3.4 Human MOG-DNA is Demethylated in the Brain and Can Be Detected by Methylation-specific Primers in Primary Oligodendrocyte Precursor Cells

**Fig. 5:**
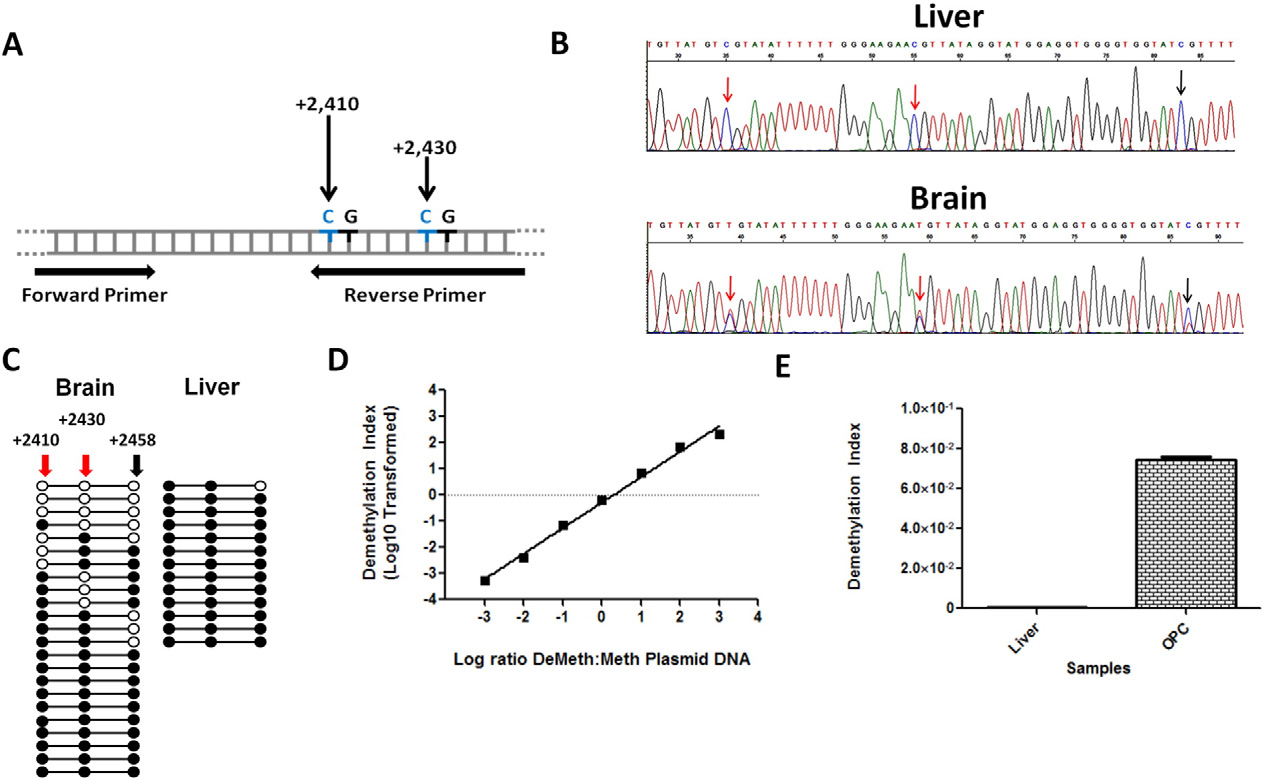
Methylation-specific primers display high specificity and sensitivity and can detect demethylated MOG-DNA in human brain and liver. A. A depiction of MOG gene region utilized for human methylation-specific qPCR analysis; cytosines at bps + 2410 and + 2430 from MOG transcription start site incorporated into reverse primer sequence. B. Sanger sequencing results of bisulfite treated DNA from human tissues. Arrows point toward CpG sites where cytosines (C) are preserved in the methylated sample (Liver), or converted to thymines (T) in a sample containing demethylated CpGs, leading to a mixed population of C′s and T's (Brain). Red arrows indicate CpGs incorporated into reverse primers. C. DNA from the brain is differentially methylated in the MOG gene compared to DNA from the liver. Sequence analysis was performed on first-step PCR product of each sample, 13 clones of liver and 23 clones of brain DNA are shown (○ represent demethylated cytosines; ●, methylated cytosines). Locations in relation to the MOG transcription start site are listed; methylation-specific human primers incorporate the CpG sites at bp + 2410 and + 2430. D. Methylation-specific primers tested using plasmids containing methylated and demethylated human MOG-DNA inserts over a wide range of serial dilutions (R^2^ = 0.992, *p* < 0.0001). E. Methylation-specific primers were used in qPCR with bisulfite treated DNA from human liver and oligodendrocyte precursor cells. Three independent analyses used to compute DMI averages.

The detection of DeMeth MOG-DNA in mouse CNS and primary ODCs suggested that MOG may be demethylated in CNS and ODCs of human origin. The demethylated CpG pairs detected in the mouse assay are evolutionarily conserved between both human and mouse (Fig. 5A). Analysis of bisulfite-treated DNA from human brain revealed the presence of demethylated CpG dinucleotides while these CpG pairs were fully methylated in DNA from human liver (Fig. 5B). This analysis was supported by cloning of purified DNA from both brain and liver which showed the presence of unique demethylation patterns on single DNA strands in the brain. These patterns were absent when liver DNA was cloned and sequenced Fig. 5C). The difference in MOG-DNA methylation between the brain and liver allowed for the design of methylation-specific primers for the human MOG gene (Table 2). Similar to the mouse assay, cloned plasmids containing the Meth and DeMeth DNA sequences of the human MOG gene were synthesized, and mixed at varied ratios over 6 logarithmic concentrations followed by analysis using methylation-specific qPCR. A positive correlation was observed between DMI level and MOG DeMeth plasmid (Fig. 5D, R^2^ = 0.992, *p* < 0.0001). Next, we compared DMI values of DNA from oligodendrocyte precursor cells (OPCs) and liver tissues. Methylation-specific primers yielded DMI values of OPC DNA that were 136-fold higher than those of liver DNA, showing a sensitivity and specificity of the methylation specific primers (Fig. 5E). These differences were further validated by comparing DMI values of plasmids containing Meth or DeMeth MOG-DNA (data not shown). Taken together, these results show the presence of differentially methylated CpG dinucleotides in the human brain and OPCs. Methylation-specific primers can successfully detect these DeMeth CpGs in DNA purified from the OPC, suggesting that this approach may be used to detect ODC death in patients with MS.

### 3.5 Circulating Free DeMeth MOG-DNA Levels Are Increased in Patients with Active Relapsing-remitting MS

**Fig. 6:**
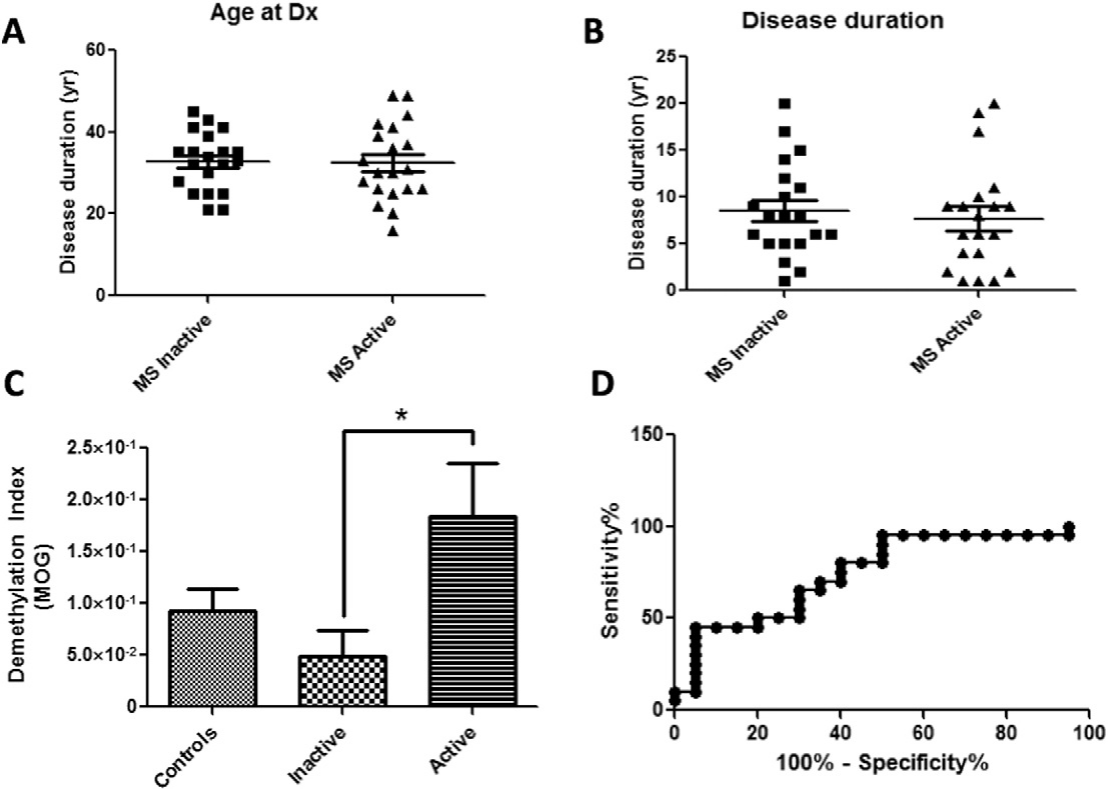
Methylation-specific primers can detect elevated levels of demethylated MOG cfDNA in patients with RRMS. A. Age at diagnosis of relapsing-remitting multiple sclerosis for both active and inactive disease groups. B. Duration of disease of relapsing-remitting multiple sclerosis for both active and inactive groups. C. Methylation-specific primers were used in qPCR with bisulfite treated DNA extracted from sera from Healthy Controls, Inactive and Active RRMS patients; ANOVA *p* < 0.029, Inactive vs. Active *p* < 0.05. D. ROC analysis of samples showed an AUC of 0.7475 with 95% confidence interval of 0.59–0.9. This analysis reached statistical significance (*p* < 0.007).

The ability of methylation-specific primers to successfully detect DeMeth MOG-DNA from OPCs suggests that this approach may be used to detect ODC-derived MOG cfDNA in patients with relapsing-remitting MS (RRMS). Serum samples from RRMS patients with active or inactive disease at the time of blood sampling were compared to sera of age and gender-matched healthy individuals for DMI values. MS patients with active and inactive disease showed similar age medians and disease duration (Fig. 6A: Age—Inactive = 40.2 ± 1.75, Active = 41.30 ± 1.5; Fig. 6B: Disease duration—Inactive = 7.7 ± 1.3, Active = 8.6 ± 1.1). DNA was purified from 300 μl of sera, bisulfite-converted, amplified by a first-step PCR reaction, and analyzed by qPCR using methylation-specific primers. DMI values were calculated based on three independent PCR reactions. DMI values of patients with active disease were 3.6 folds higher than inactive disease showing statistical significance (Fig. 6C, DMI—Healthy controls = 9.23 × 10^− 2^ ± 2.16 × 10^− 2^, Inactive MS = 4.89 × 10^− 2^ ± 2.49 × 10^− 2^, Active = 18.36 × 10^− 2^ ± 5.18 × 10^− 2^. ANOVA *p* < 0.029; Inactive vs. Active *p* < 0.05). ROC analysis of samples showed an AUC of 0.7475 with 95% confidence interval of 0.59–0.9. This analysis reached statistical significance (Fig. 6D, p < 0.007). Taken together, our data show, for the first time, the utility of MOG cfDNA to detect active MS in patients with RRMS.

## 4 Discussion

Differentially methylated cfDNA can be used as a biomarker of cancer and autoimmune diabetes. Here we report the presence of differentially methylated CpG dinucleotides in the coding region of the MOG gene in ODCs. These DeMeth CpGs are detected by methylation-specific primers and measure the presence of ODC-derived MOG cfDNA in the blood of mice with widespread demyelination. Moreover, methylation-specific primers for human MOG-DNA can detect increased ODC loss in the sera of patients with active RRMS when compared with inactive disease and healthy controls. Our results describe a new assay for measuring differentially methylated MOG cfDNA as a minimally invasive biomarker of ODC loss in RRMS. A biomarker capable of measuring ODC loss may advance our ability to better diagnose MS progression in conjunction with current imaging techniques, allow for better disease prognosis and modeling of ODC cell loss, and for measuring clinical efficacy of novel therapeutic agents.

MOG expression is found predominantly in ODCs (Coffey and McDermott, 1997, Solly et al., 1996). Recent reports suggest that methylation may play an active role in the transcription of MOG as the expression of methyl-CpG binding protein 2 can bind to Meth CpGs and inhibit the expression of several myelin components, including MOG (Solly et al., 1996). Despite these findings, little is known about the state of MOG-DNA methylation and the role methylation plays in gene regulation. The data presented above show the presence of several demethylated CpGs in DNA from primary mouse ODCs and primary human OPCs when compared with other tissues. These demethylated dinucleotides were absent in other cell types and tissues and are evolutionarily conserved in the MOG coding region. While our data does not address the role of methylation in the regulation of MOG gene expression, our findings provide a strong support for it in both murine and human cells. The presence of DeMeth CpG pairs in MOG-DNA from ODCs provided an opportunity to design methylation-specific primers for the detection of ODC-derived DNA. This approach was previously used to detect the loss of insulin producing β-cells in patients and animal models of type 1 diabetes (Akirav et al., 2011, Olsen et al., 2016) and for identifying cell loss in MS (Lehmann-Werman et al., 2016). Methylation specific primers showed a high degree of specificity for Meth or DeMeth MOG-DNA when tested on both artificially synthesized DNA and DNA from ODC and brain origin, suggesting that these primers may detect as little as 1 copy of DeMeth DNA in 1000 copies of Meth MOG-DNA.

The fact that these primers were able to detect DeMeth MOG-DNA in mice injected with artificial DNA confirmed the ability of the assay to detect ODC-like DNA in vivo. To further test the ability of the primers to detect ODC cell loss, we used the cuprizone model of ODC-toxicity and widespread demyelination in mice. In previous reports, ODC gene expression in the brain, used as an indicator of ODC function and mass, was reduced early during treatment, with the reemergence of MAG-expressing cells in the brains of cuprizone-treated mice from week 6 onwards post treatment (Lindner et al., 2008, Praet et al., 2014). Additional studies report complete remyelination in the cuprizone mice upon removal of the cuprizone diet. The remyelination was complemented with the detection of several myelin proteins, excluding MOG, whose expression was delayed for several weeks (Lindner et al., 2008, Praet et al., 2014). Indeed, Methylation-specific primers detected ODC-cell loss as early as 3 weeks post cuprizone treatment, with the highest increase detected at Day 14 post treatment. Moreover, our results showed relatively low levels of ODC-derived DeMeth MOG-DNA in the blood of mice from week 5 until week 8 post-treatment, corresponding to previous reports showing the reemergence of ODCs in the brain. Our overall findings suggest that ODC loss can be detected during the acute or toxic phase of cuprizone administration while in later time points the ablation of ODCs resulted in reduced ODC-derived DNA in the blood.

The detection of ODC-derived DeMeth MOG-DNA in the cuprizone mouse model lead us to examine the methylation state of the MOG gene in human DNA. Analysis of an evolutionarily conserved region of MOG-DNA in human brains and liver showed an identical demethylation pattern in CpG dinucleotides also present in mouse MOG. The fact that sequencing analysis of brain DNA revealed only a partial conversion of C → T suggests that non-ODC cells contribute to the Meth form of MOG-DNA while ODCs are the sole source of DeMeth MOG-DNA as seen in the O4^+^ and O4^−^ cell fractions in the mouse. Methylation-specific primers designed to distinguish between the Meth and DeMeth form of CpGs + 2410 and + 2430 showed a high degree of specificity and sensitivity similar to that of the mouse assay, and were able to detect DeMeth MOG in brain DNA at DMI levels nearly 10,000 folds higher than those observed in the liver. When tested, these primers showed a high DMI signal in OPCs when compared with liver, showing the ability of the assay to detect ODC-like DNA.

The high degree of sensitivity and specificity of the human methylation-specific primers allowed us to analyze blood samples from 40 patients with RRMS, with active (*n* = 20) or inactive (*n* = 20) disease. Healthy controls (*n* = 20) were also used to establish the baseline background DMI levels of normal subjects. Disease activity in RRMS patients was determined by clinical episodes and, in some cases, by the formation of new brain lesions in the CNS. While both RRMS patients with inactive and active disease were similar in age, gender, and duration of disease, patients with active RRMS had significant elevation in DMI values. ROC analysis of active and inactive RRMS revealed average AUC and preliminary cutoff values of specificity and sensitivity for assay performance. Additional studies consisting of larger patient cohorts would be needed to validate these values and allow for their use in the clinic. To our knowledge, this is the first report demonstrating the utility of differentially methylated ODC-derived cfDNA as a biomarker of cell loss in RRMS where DMI levels reflect disease activity.

This report describes the differential methylation of the MOG gene in ODCs and brain tissues of both mouse and human origin. The unique DeMeth patterns in ODCs provide a new biomarker for the detection of ODC-cell loss in MS. Currently, diagnosis of MS is based on several criteria (Polman et al., 2011), including identification of lesions by MRI and positive identification of biomarkers in cerebral spinal fluid (CSF). MS is a financially burdening disease (Adelman et al., 2013, Kobelt et al., 2006), making MRIs cost-prohibitive for many underinsured patients. Despite the ongoing identification of useful biomarkers in CSF testing (Teunissen et al., 2015, Fitzner et al., 2015), lumbar punctures are an invasive procedure which prevents collecting samples from healthy individuals. The collection of cfDNA is a cost effective, minimally invasive procedure that can be implemented for diagnosis, prognosis, and monitoring of treatment efficacy. The ability to measure ODC loss may prove beneficial not only to MS but also to other disease afflicting ODC survival such as progressive multifocal leukoencephalopathy (PML).

The following is the supplementary data related to this article.

Supplemental Fig. 1 Sanger sequencing results of magnetic-beads enriched O4-positive and O4-negative cells from mouse brains. Black arrow point to differentially methylated CpG identified following bisulfite treatment. Images represent one repeat of four independent preparations. Supplemental Fig. 1

## Funding

This work was supported by the National Multiple Sclerosis Society (Grant No. PP2130), the Marilyn Hilton Award for Innovation in MS Research, and Winthrop University Hospital Pilot Award. Granting agencies did not play a role in the design, data collection, or analysis of data.

## The Duality of Interest

The authors declare that there is no duality of interest associated with this manuscript.

## Author Contributions

EMA and JAO conceived and designed the experiments. JAO, LAK, RCT and EMA performed the experiments. JAO and EMA analyzed the data. JAO, LAK, MGS, MMS and EMA wrote the paper.

## Figures and Tables

**Table 1:** Primer sequences and PCR protocols for mouse MOG analysis.

PCR type	Primer designation	Primer sequence 5′ → 3′	Product length	PCR protocol
First-step PCR	Forward	GAGTGATAGGATTAGGGTATTTTATT	169 bp	50 cycles, annealing temperature 57 °C
Reverse	TCTACATCTTAATCCTTACCATTTC
Methylation-specific nested qPCR	Common forward	GAGTGATAGGATTAGGGTATTTTATT	87 bp	40 cycles, annealing temperature 64 °C
Hypermeth-specific reverse	TAACGTTCTTCCCAAAAAATATACG
Hypometh-specific reverse	TAACATTCTTCCCAAAAAATATACA
Native sequence first-step PCR	Forward	GAGTGATAGGACCAGGGTATCCCATC	169 bp	Cycles no. variable, annealing temperature 57 °C
Reverse	CTGCATCTTGGTCCTTGCCATTTC

**Table 2:** Primer sequences and PCR protocols for human MOG analysis.

PCR type	Primer designation	Primer sequence 5′ → 3′	Product length	PCR protocol
First-step PCR	Forward	GGGTAGTTTAGAGTGATAGGATTAAGATAT	152 bp	50 cycles, annealing temperature 57 °C
Reverse	TAAAAATAAACCACCCTAAAAAAAA
Methylation-specific nested qPCR	Common forward	GGGTAGTTTAGAGTGATAGGATTAAGATAT	97 bp	40 cycles, annealing temperature 64 °C
Hypermeth-specific reverse	TAACGTTTTTCTCAAAAAATATACG
Hypometh-specific reverse	TAACATTCTTCCCAAAAAATATACA

**Table 3:** Demographic and disease history for RRMS and control subjects.

Parameters	Groups		
Ctrl	RRMS	
Inactive	Active
N (all females)	20	20	20
Ethnicity (W/AA)	20/0	19/1	20/0
Age (years)	41.3 ± 1.5	40.20 ± 1.75	41.3 ± 1.5
Age at dx (years)	–	32.5 ± 2.1	32.8 ± 1.6
Disease duration (years)	–	7.7 ± 1.3	8.6 ± 1.1
aDuration from last epi (years)	–	1.5 ± 0.5	–
bTime between epi (years)	–	1.9 ± 0.4	1.6 ± 0.6

a 18/20 patients reporting.

b 29/40 patients reporting.
