# Supplementary material for: A Minimally-invasive Blood-derived Biomarker of Oligodendrocyte Cell-loss in Multiple Sclerosis
Source: eBioMedicine. 2016 Jun 27;10:227–35. doi: 10.1016/j.ebiom.2016.06.031 (PMC5006601; doi:10.1016/j.ebiom.2016.06.031)
Supplement: Supplemental Fig. 1 — Sanger sequencing results of magnetic-beads enriched O4-positive and O4-negative cells from mouse brains. Black arrow point to differentially methylated CpG identified following bisulfite treatment. Images represent one repeat of four independent preparations. [file mmc1.pptx]

## Slide 1
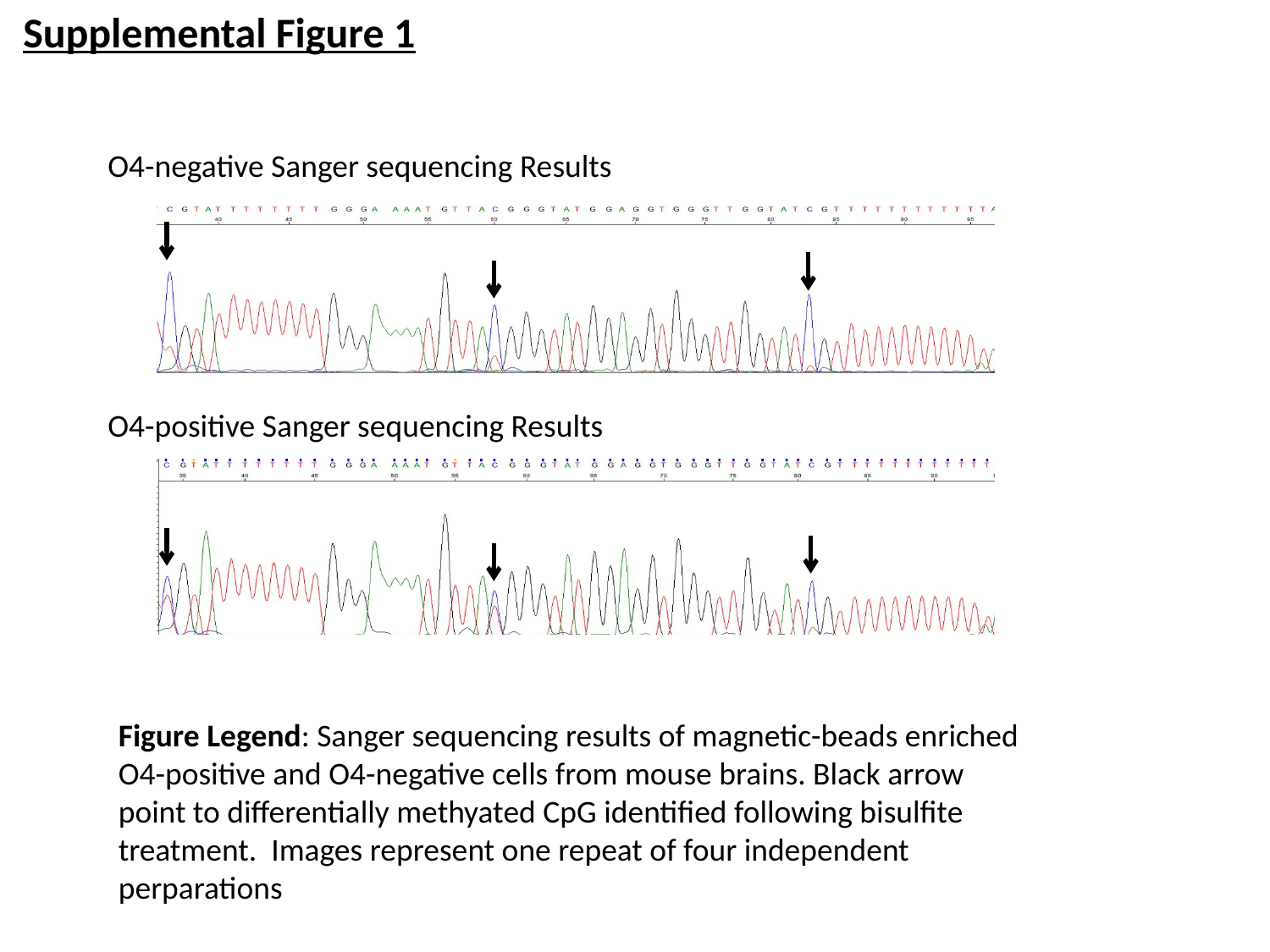

Supplemental Figure 1
O4-negative Sanger sequencing Results
O4-positive Sanger sequencing Results
Figure Legend: Sanger sequencing results of magnetic-beads enriched O4-positive and O4-negative cells from mouse brains. Black arrow point to differentially methyated CpG identified following bisulfite treatment. Images represent one repeat of four independent perparations
